# A Multi-Component Educational Intervention for Addressing Levels of Physical Activity and Sedentary Behaviors of Schoolchildren

**DOI:** 10.3390/ijerph20043003

**Published:** 2023-02-09

**Authors:** Ana Vilardell-Dávila, Gloria Martínez-Andrade, Miguel Klünder-Klünder, América Liliana Miranda-Lora, Eugenia Mendoza, Samuel Flores-Huerta, Jorge Eduardo Vargas-González, Ximena Duque, Jenny Vilchis-Gil

**Affiliations:** 1 Epidemiological Research Unit in Endocrinology and Nutrition, Hospital Infantil de México Federico Gómez, Ministry of Health (SSA), Mexico City 06720, Mexico; 2 Mexican Academic Area of Nutrition, Institute of Health Sciences, Autonomous University of the State of Hidalgo, Pachuca 42039, Mexico; 3 Deputy Director of Research, Mexico Children’s Hospital Federico Gómez, Mexico City 06720, Mexico; 4 Unit of Medical Research in Infectious and Parasitic Diseases, Mexican Institute of Social Security (IMSS), Mexico City 06720, Mexico

**Keywords:** educational intervention, obesity, physical activity, schoolchildren, sedentary behaviors

## Abstract

Physical inactivity and a sedentary lifestyle are risk factors for excess weight and obesity in childhood. It is, therefore, necessary to adopt strategies which can modify these behaviors during childhood, the age at which habits are formed. This study aimed to evaluate the impact of an educational intervention using digital media and face-to-face activities involving children, parents, and the school community on the level of physical activity and sedentary behavior among schoolchildren. This was a secondary analysis of data obtained from a community trial in which students from four primary schools in Mexico City participated. Two schools were assigned to the intervention group (IG) and two to the control group (CG). The intervention lasted 12 months and included a face-to-face component, which involved sessions and workshops for parents and children, as well as visual material for children and a distance component utilizing electronic means (web portal and text messages to mobile phones) for parents. Anthropometric measurements were taken and information was collected on moderate to vigorous physical activity performed by the children and on the time that the schoolchildren spent in front of screens at the beginning of the study and at 6 and 12 months. Information on 201 children from the IG and 167 children from the CG was included in the analysis. At 12 months, the IG showed a mean decrease of 33.4 min/d [95% CI: −53.5 to −13.3] in screen time, while the CG showed an increase of 12.5 min/d [CI 95%: −10.5 to 35.6], *p* = 0.003. After 12 months of follow-up, applying this educational intervention reduced the time that schoolchildren spent in front of screens. Educational intervention is a feasible and accessible strategy for promoting changes in sedentary behaviors in the school-age population.

## 1. Introduction

Childhood obesity constitutes a public health problem because of its high prevalence worldwide and the severity of its comorbidities, which include metabolic syndrome, type 2 diabetes, high blood pressure, dyslipidemia, obstructive sleep apnea, and nonalcoholic fatty liver [1]. The etiology of excess weight and obesity is associated with several genetic and environmental factors, and physical inactivity and sedentary lifestyle are two of the most recognized within the latter category [2]. Physical inactivity is considered to be this century’s greatest public health problem [3,4] and implies a lack of compliance with the minimum international physical activity (PA) recommendations needed to maintain a satisfactory state of health. Additionally, sedentary behaviors involve a lack of physical movement during waking hours, which also includes time spent sitting or lying down, where energy expenditure is close to that of rest (1 metabolic equivalent for task (MET)). This results in minimum energy expenditure [5,6]. In the young population, low levels of physical activity and high levels of sedentary behaviors have been associated not only with overweight and obesity, but also with important morbidity, such as prehypertension or even hypertension [7]. Physical inactivity and sedentary behaviors are independent risk factors for the development of obesity, and they also increase overall morbidity and mortality; therefore, they must both be addressed simultaneously [5,6,8].

Globally, Mexico is among the countries with the highest rates of overweight and obesity in childhood. In the National Health and Nutrition Survey (ENSANUT) of 2020, a combined prevalence of excess weight and obesity of 38.2% was identified among the population aged between 5 and 11 years [9]. According to the ENSANUT of 2016, only 17.2% of the population between the ages of 10 and 14 years met the World Health Organization’s (WHO) PA recommendation, while 71.7% reported more than 2 h per day in front of screens, one of the primary indicators of sedentary behavior [10,11]. This suggests the need for interventions aimed at modifying these habits in the Mexican population beginning at an early age.

Healthy eating and physical activity habits are learned and fostered at home; parents have a transcendental role in promoting participation in sports and active lifestyles in children and adolescents [12,13]. For this reason, educational interventions aimed at improving children’s eating habits and PA levels have become increasingly focused on the family [13,14]. However, schoolchildren may be more receptive to educational intervention than parents, and even more so when part of the intervention takes place within the schools’ environment with their peers. Interventions whose main objective is to achieve health behavior change for reducing the burden of preventable disease should use more than one communication channel and involve the main social agents that could influence the change sought [15]. Internationally, educational intervention models have been proposed to modify dietary habits and PA in schoolchildren and adolescents, but the results have been heterogeneous. Informative and cognitive, behavioral, environmental interventions, social support strategies, etc. have been proposed, emphasizing the fact that it is difficult for an individual to change habits just because they are informed about the need to do so and, therefore, that it is necessary to employ integrative strategies that promote behavioral change [16]. Integrative interventions at different levels of influence are necessary when lifestyle changes are sought [15]. Studies of intervention have been implemented to change lifestyles of eating and physical activity; however, many have been conducted without a control group [17,18], for short periods [19], and have had no lasting effect on nutritional status or lifestyles [20,21]. 

Therefore, greater efforts are needed to promote PA through research and the implementation of interventions, which may subsequently support the development of policies aimed at addressing this health problem. 

Systematic reviews that have assessed educational interventions in children have determined that those that modify habits are characterized by including the following items in their planning: social support of the participant, greater parental involvement, and parental responsibility for carrying out the intervention with their children [22,23]. Other authors have concluded that directing the components of educational interventions solely toward the school environment may be insufficient to cause a real public health population level impact in terms of modifying habits in children. Thus, the additional need to direct interventions toward the home context is noted [24,25]. The ecological model of health–disease posits that the environment is a crucial factor in the development of the majority of diseases, specifically chronic noncommunicable diseases. This environmental influence begins within the family context and expands to the school, community, and the national context, including health policies [15,26]. In this study, the intervention was aimed at raising awareness among the participants and their families about the importance of performing more physical activity and reducing the level of sedentary behaviors. Therefore, home and school influence the formation of habits in children and adolescents, so it is essential to include them when developing educational intervention models. Thus, this study aimed to evaluate the impact of an educational intervention based on the use of digital media and face-to-face activities involving children, parents, and the school community over the PA and sedentary behavior among schoolchildren in Mexico City.

## 2. Materials and Methods

### 2.1. Study Design and Population

This study is part of a community trial whose main purpose was to evaluate the effect of a multi-component educational intervention aimed at schoolchildren and their parents. The intervention consisted of promoting healthy eating habits, increased physical activity, and decreased time spent in sedentary behavior in schoolchildren. The design and implementation of the intervention have been previously described [27,28]. The data analyzed in this study come from the original study, which was carried out from October 2013 to July 2014.

Four primary schools located in the center of Mexico City with similar number of students were included in the study; two were public schools and two were private. One public and one private school were assigned to the intervention group (IG), and one public and one private school were assigned to the control group (CG). Schoolchildren in grades 1 through 4 (6 to 10 years) and their parents were asked to participate in the study. Before the study implementation, educational materials and the website were designed and developed, which were the tools of the intervention. Prior to beginning the study, the objectives of the study, procedures, and measurements to be carried out during the study’s one-year duration were explained to the parents, children, and teachers. Parents and children signed an informed consent and an assent agreeing to participate in the study, respectively. Boys and girls were included, regardless of their z-score for body mass index (BMI). Children who were participating in a weight reduction program were excluded. Participants who did not answer the baseline questionnaire on PA and sedentary behaviors were removed from the analysis. The study was approved by the Ethics, Research, and Biosafety Committees of the Federico Gómez Children’s Hospital of Mexico. Additionally, the Ministry of Public Education and directors of the participating schools authorized the study.

### 2.2. Study Groups

In the IG, a multicomponent intervention known as ALAS (Aliméntate y Actívate Sanamente or “Eat and activate yourself healthily”) was implemented from October 2013 to July 2014. The intervention involved children and their parents from participating schools [18]. The parent-oriented components included (1) distance education material provided through a website installed on the hospital webpage (http://himfg.com.mx/interna/dirinvestiga/alas.php), which was updated every two weeks (20 topics in total). In short, the website contained weekly topics on healthy eating and physical activity and advice on reducing a sedentary lifestyle, which could be accessed at any time; (2) text messages (one per week, 40 in total) about healthy eating, PA, and sedentary behaviors, which was sent to the parents’ mobile phones, the message was related to the current topic of the website that motivated and reinforced behavior changes; and (3) three face-to-face informative sessions of 1 h each, once every 2 months, for the purpose of giving them feedback on topics of eating and physical activity that were on the website or to participate in some project activities; in the sessions were delivered two printed guides with recommendations to improve eating habits and PA and to avoid sedentary behavior. The children’s components were (1) four face-to-face workshops given by nutritionists and a physical educator, 1 every 2 months, with a duration of 1.5 h. The workshops were integrated with both board games and physical games and with educational materials to reinforce healthy eating habits and physical activity; (2) graphical educational material that consisted of the Mexican Eatwell Plate and the Pyramid of PA (Figure S1); and (3) an invitation to visit the “Health and Life in Balance” room of the Universum Museum of Sciences of the National Autonomous University of Mexico. Finally, posters were placed throughout different areas of the schools monthly. All the components mentioned provided information on healthy eating, the promotion of PA, and the prevention of excessive sedentary behavior during the day. The components of the intervention have been described in detail previously [27,28].

The educational materials of the different components promoted a less sedentary lifestyle and increased PA and contained messages aimed at encouraging active play, participating in sports for at least 1 h daily, walking as a way to get from place to place, and decreasing screen time to less than 2 h daily (Figure S1).

Results of nutritional status assessments obtained through anthropometric and biochemical measurements were provided to all participants. The CG also received general recommendations on how to improve their eating and PA habits; this information was delivered in two printed sheets only at the beginning of the study. The recommendations focused on giving short messages about breakfast habits, school lunch, consumption of fruits, vegetables, and natural water, and limiting the consumption of sweet drinks; likewise, children were encouraged to perform physical activity as a family, help with chores from home, and walk instead of using transportation. In addition, it was recommended to reduce the time in front of screens. This group received the same educational intervention received by the IG for one year after the end of the study. However, the evaluation of impact of this intervention in the CG was not part of the study follow-up.

### 2.3. Measurements

Parents answered a questionnaire adapted from a validated Activity and Inactivity of Mexican Students (CAINM) questionnaire [29]. The questionnaires were provided to the schoolchildren so they could give them to their parents at baseline, and then again at 6- and 12-month follow-ups.

The parents stated the type of exercise or sports activities performed by their children outside of school activities, the frequency (days per week), and the average duration of each session. This information was analyzed based on the categories of PA and type of moderate or intense exertion according to the Youth Compendium of Physical Activities developed by the Youth Energy Expenditure Workgroup of the National Collaborative on Childhood Obesity Research (NCCOR) in the United States [30]. In this compendium, values corresponding to different categories of PA are presented in METy. METy values represent METs adjusted to correspond to specific metabolic and physiological characteristics for four age groups during childhood and adolescence (6–9 years, 10–12 years, 13–15 years, and 16–18 years of age); the METy were calculated using the ratio of the activity-specific metabolic rate relative to the estimated basal metabolic rate for that age group. 

If the activity reported by the parents corresponded to a METy value ≥3.0 (for example, soccer, swimming, or basketball), it was considered to be within the category of moderate and vigorous PA (MVPA). The minutes involved in these activities were added to obtain the total weekly value of AFMV minutes. Additionally, a dichotomous variable was generated to identify whether or not participants performed some MVPA weekly. Likewise, the total PA time (TPA) was calculated by adding the MVPA time and time spent involved in light-intensity PA (e.g., walking). PA performed during physical education class (50 min weekly), which was part of the school curriculum, was not included, because the children in the control and intervention group had the same time dedicated to physical activity in a week within school hours.

The time-in-front-of-a-screen variable was constructed to assess the sedentary behavior of the children. The daily amount of time that participants spent watching or looking at TVs, computers, video games, and cell phones were added. A dichotomous variable was also generated that identified whether schoolchildren spent ≤120 min/d or >120 min/d of screen time [31,32].

Additionally, sociodemographic characteristics were recorded in a questionnaire at the beginning of the study. The educational level of mothers was categorized as primary or secondary education, preparatory or technical school, and university or postgraduate degree. The socioeconomic level was constructed based on information on the assets that the family owned in their home (e.g., telephone, internet, washing machine, car, etc.). It was analyzed using principal components analysis, and a score was obtained according to the number of assets [33]. This score was then classified into tertiles to assess the households’ socioeconomic level.

Anthropometric measurements were made at the beginning of the study in accordance with international procedures [34]. Weight (kg) was determined using a digital scale (SECA model-882, SECA Corp., Hamburg, Germany) with a precision of 0.1 kg. A stadiometer (SECA model-225, SECA Corp., Hamburg, Germany) with 0.1 cm precision was used to measure height (cm). The z-score for body mass index (BMI) was calculated using schoolchildren’s weight, height, age, and gender, considering the WHO standards [35]. The children were classified as being normal weight (Z-score −2 to <1), overweight (Z-score 1 to <2), or obese (Z-score ≥ 2).

### 2.4. Statistical Analysis 

Descriptive statistics were used to compare the characteristics of the study groups. The Mann–Whitney U test was used to compare quantitative variables without normal distribution between groups at baseline. Categorical variables were compared between groups using Pearson’s chi-squared test.

The change in the number of minutes of MVPA, TPA, and time in front of screens was obtained for both groups from baseline to 6 months and from baseline to 12 months. Intragroup changes were compared using Wilcoxon’s or Student’s *t*-tests for paired data. The Mann–Whitney U test was used to compare median changes in order to assess the differences between the IG and CG. Only the change in screen time from baseline to 12 months followed a normal distribution; therefore, the comparison between groups of this variable was conducted using the Student’s *t*-test for independent data. The equal proportions test was used to assess the changes in MVPA frequency and screen time from baseline to 6- and 12-month follow-ups. Linear regression models were used to determine the change in screen time from baseline to 12 months, taking into consideration the sociodemographic variables. However, no variable was found to be a statistically significant predictor of the change in screen time at end of study, so these models are not shown.

The analyses were performed using Stata v14.0 (Stata Corp., College Station, TX, USA). *p* values < 0.05 were considered to be statistically significant for all analyses.

## 3. Results

### 3.1. General Characteristics of the Study Population

**Figure 1:**
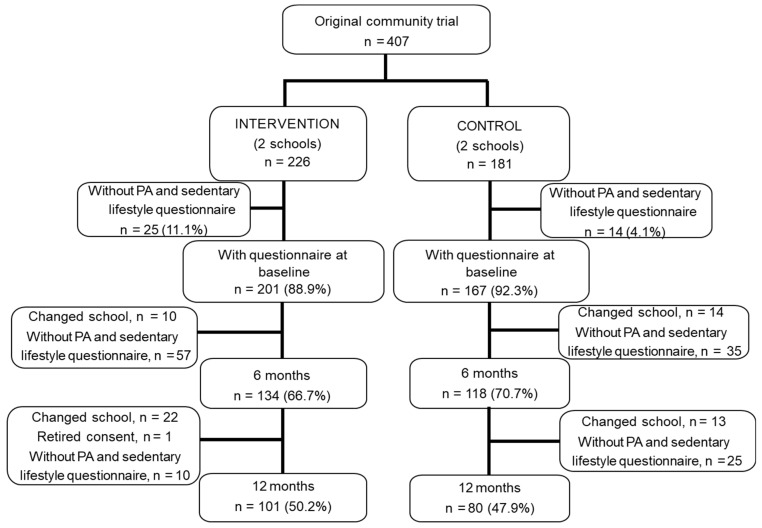
Flow of study participants.

The study population consisted of 368 children, which was 90.4% of the sample population that participated in the original community trial and whose parents answered the baseline questionnaire on PA and sedentary behaviors. Of these, 201 belonged to the IG and 167 belonged to the CG (Figure 1). A total of 101 (50.2%) children in the IG and 80 (47.9%) children in the CG completed the 12-month follow-up. Those missing from the follow-up were mainly due to children changing schools or parental nonresponse to the follow-up PA and sedentary behavior questionnaires. Figure 1 shows the flow of participants throughout the study.

The differences between participants who completed (n = 181) and did not complete (n = 187) the 12-month follow-up were determined. Differences were found in terms of the percentage of male participation by group during follow-up, 46.7% in the group that completed follow-up vs. 59.8% in the group that did not complete follow-up (*p* = 0.001). Additionally, the children who completed follow-up had a higher percentage of two-parent families vs. the children who did not complete follow-up (73.1% vs. 64.6%; *p* = 0.031) (data not shown). No differences were observed in the remaining variables.

Table 1 shows the characteristics of the participants according to the group to which they were assigned. Distribution according to age, gender, and anthropometric characteristics was similar in the two groups. Some socioeconomic and technological access differences were identified between the groups. In the IG, a higher proportion of participants were from public schools, a higher frequency had low and medium socioeconomic status, a lower proportion were single-parent families, and a higher frequency of mothers possessing a cell phone and a higher frequency of internet access at work were all observed.

### 3.2. Physical Activity

**Figure 2:**
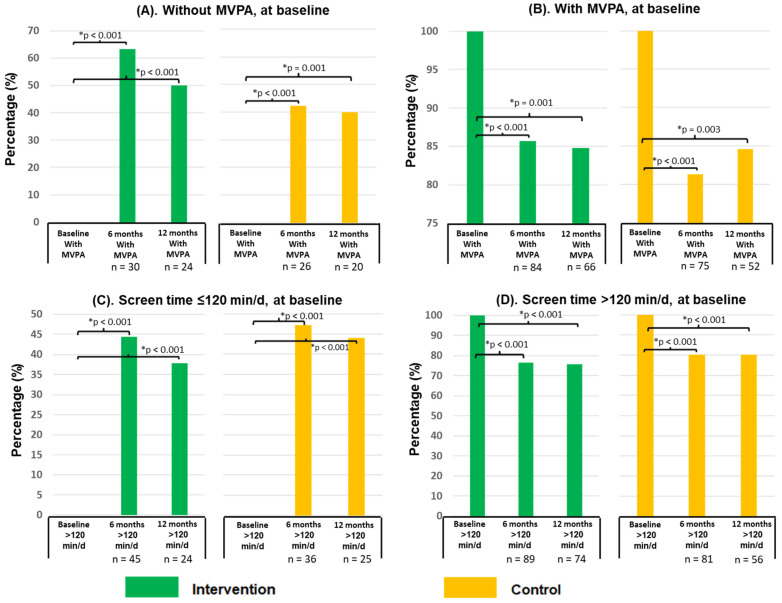
Stratified analysis of the effect of the intervention according to the performance at the beginning of the study or not of MVPA and the amount of time in front of screens. MVPA: moderate to vigorous physical activity; * test for equality of proportions.

Table 2 shows the changes in MVPA and TPA at the 6- and 12-month follow-ups for both study groups. No significant differences were found within or between the groups. Figure 2A,B show the frequency changes among schoolchildren who did or did not perform MVPA at the beginning of the study. An increase of 40% and 60% in the CG and IG, respectively, was observed during follow-ups in those who did not perform MVPA at the beginning of the study (*p* < 0.001), but there were no significant differences between the study groups. Conversely, in those who reported performing MVPA at the beginning of the study, a decrease of approximately 15% (*p* < 0.001) was observed during the follow-up period, with no differences observed between the groups. 

### 3.3. Screen Time

Table 3 shows the changes in screen time (min/d) at 6- and 12-month follow-ups. A mean reduction of 33 min/d in screen exposure time was seen in the IG (*p* = 0.001) at 12 months, while no significant change was seen in the CG. A difference of 45 min/d in mean screen time was observed between groups at 12 months (*p* = 0.003). Changes in the proportion of participants spending ≤120 or >120 min of screen time at baseline are shown in Figure 2C,D. Increases of 38% and 48% in the IG and CG, respectively, were observed during follow-up in children who spent ≤ 120 min of screen time at baseline (*p* < 0.001), but no significant differences between study groups were observed. Conversely, during the follow-up period, a decrease of approximately 20% (*p* < 0.001) was observed among those who reported having >120 min of screen time at the start of the study, with no differences observed between groups.

### 3.4. Parental Exposure to Educational Material

Additionally, parental exposure to educational material in the IG was evaluated. Regarding the face-to-face information sessions, 43.3% (n = 87) of parents did not attend any of the sessions, 37.8% (n = 76) attended only one, 16.8% (n = 34) attended two, and 2.0% (n = 4) attended the three sessions that were held during the intervention. The web portal was consulted at least once by 43.3% of parents (n = 87) and 99.5% of parents received the weekly text messages sent to their mobile phones.

The data were analyzed in relation to parental exposure to the educational material. IG participants whose parents attended at least one face-to-face session, as well as those who consulted the website, experienced a significant decrease in screen time of approximately 45 min/d at the 6- and 12-month follow-ups (*p* = 0.001). Conversely, there were no significant changes in the children of parents who did not attend the sessions or did not consult the website (Tables S1 and S2). 

## 4. Discussion

This study reports the results of a multicomponent educational intervention on the level of PA and sedentary behavior among schoolchildren based upon the use of digital media and face-to-face activities with the schoolchildren and their parents, and the school community in general, along with the use of visual material at school. The results indicate that a reduction in the average exposure to screen time was achieved at the end of the intervention with one year of follow-up. This reduction was more significant in participants whose parents were more involved with the intervention. This could indicate two relevant factors: (1) the importance of involving the families of schoolchildren in educational activities which are aimed at behavioral changes in support of a healthy lifestyle; and (2) once the message has been delivered, behavioral changes leading to a healthy lifestyle can be achieved. Regarding the latter, a multi-component strategy with various communication channels, such as that used in this study, increases the probability of exposure to information promoting a healthy lifestyle. However, the intervention did not show an effect on the minutes/week that schoolchildren spend performing MVPA; perhaps it would be necessary to include the participation of the school so that physical exercise and sports activities can be offered as extracurricular activities as a complement to the educational intervention. 

Several interventions have been proposed that are aimed at changing the habits related to sedentary behaviors to prevent excess weight and obesity in children. In this study, an intervention with multiple components achieved a decrease in screen exposure time, achieving a reduction of approximately half an hour per day in the IG. This result is consistent with those reported by other authors who found reductions of between 5 and 60 min/d [16]. However, it has been determined that the interventions that tend to be most successful in modifying these habits in adults are those whose educational components are developed to a specific behavior, instead of multiple behaviors that include diet, PA, prevention of sedentary lifestyle, etc. [36]. On the other hand, in this study, a nonsignificant increase in the time spent in front of screens in the control group was observed; it could be explained by age; studies have observed that, as age increases, time spent in front of screens tends to increase as well [37]. So, the increase in screen time in the control group participants might be what would normally be expected as a child approaches adolescence. 

The present study’s baseline data show that the schoolchildren spent an average of 180 min/d watching screens. There is no currently defined consensus on the maximum period of screen time to which school-age children can be exposed without presenting changes in their health. The American Academy of Pediatrics previously established a recommendation that children older than 2 years should not spend more than 2 h a day in front of screens. Currently, the importance of creating a plan for the use of digital media, personalized for each family, and established according to the characteristics, needs, and values of the family is stressed [38]. It has been demonstrated that excessive exposure to screens can have repercussions on various aspects of health during childhood. Children who spend ≥ 5 h/d watching TV are five times more likely to be overweight than those who spend ≤2 h/d [39]. It has also been reported that children 4 to 9 years of age who spend more than 1.5 h/d watching TV have an increased risk of developing obesity [40]. For every additional hour that children spend in front of screens, there is a lower probability that they will consume fruits or vegetables and engage in PA, and a greater probability that they will consume junk food and be overweight or obese [41]. Regarding other health-related aspects, the excessive use of digital media and time in front of screens has been associated with sleep disorders [42], as well as with psychological and cognitive disorders, including depressive syndromes, anxiety, hyperactivity, and inattention [43]. In a study involving young people from Hong Kong with a severe form of social isolation known as “hikikomori”, in this lifestyle, young people spend their time at home. The participants in this study were 13 and 34 years old; two groups were identified, those who have lived more than 6 months in an asocial and sedentary lifestyle and those who have lived in this behavior for less than 6 months. A high prevalence of hypertension (15.4%) and prehypertension (31.7%) was found, the prevalence of prehypertension was higher in cases of 6 months or more in this lifestyle compared to cases of recent onset (38.5% vs. 25.0%); the prevalence of overweight/obesity was also higher in cases of more than 6 months with this behavior (48.1% vs. 19.2%); the participants’ diet was unhealthy, with high consumption of fast food, sugary drinks, and only one or fewer servings of fruits and vegetables per day [7]. These results show that a sedentary lifestyle and social isolation with increased exposure to screen time are associated with health risks.

In this study, the children performed MVPA 150 min/week on average during follow-up. Therefore, the WHO’s recommendation to perform MVPA for at least 60 min/d [4] was not met. However, it is noteworthy that this reported activity included only what the children did outside of school activities. The results reported by other authors regarding changes in PA through educational interventions are very diverse. Some authors have not found a satisfactory effect over different follow-up periods [44,45,46], while others have found very varied effects, ranging from 2.6 to 283 min/d in the total PA time [47]. Additionally, ranges of increase from 5 to 45 min/week have been reported for MVPA [24]. Even though no differences were observed in MVPA between groups in this study, it was possible to determine a differential effect resulting from the intervention in the stratified analysis according to whether or not they performed MVPA at baseline. Among those who did not report performing MVPA at the beginning of the study, there was an increase in the frequency (40–60%) of participants who performed MVPA at the 6- and 12-month follow-ups. Conversely, it was reported that about 15% of participants who performed MVPA at baseline stopped performing this type of activity at 6 and 12 months in both groups. A possible reason for this finding is a probable overestimation of the time spent in MVPA or of the intensity of the activity that they performed at the beginning of the investigation. This could have changed after exposure to the educational material. This is one of the limitations of MVPA evaluation through the use of questionnaires. A study that compared the original questionnaire on which the one used in this study was based with accelerometry data found that MVPA was overestimated by the questionnaire and that mild-intensity PA, along with sedentary behaviors, were underestimated [48]. More research on strategies to increase the physical activity that children perform is necessary. The relationships between physical activity, cardiovascular health, and body composition have been well established. The physical activity in children plays an important role in cardiovascular, mental, and musculoskeletal health and physical, social, and cognitive development [49]. The health professionals should play a critical role in encouraging physical activity in children and their families through physical literacy, providing guidance toward meeting recommendations, setting examples, and incorporating physical activity prescription in the clinical and community setting [49].

Varied results have been reported regarding the use of digital media as part of educational interventions aimed at changing habits. Knowlden et al. used a web portal to transmit information to mothers and the observed decrease was not significant between groups, even though there was an overall decrease of 27.6 min/d in screen time [50]. Baranowski et al. also used a web portal to send educational information to girls and their parents but found no effect on their sedentary behavior from the intervention [51]. De Lepeleere et al. employed educational videos to convey information to parents of schoolchildren and found no effect on screen time [52]. In contrast, using mobile phones in low-income countries, such as India, has been shown to be an effective method of promoting maternal and child health in strategies such as the Healthphone Initiative [53]. In our investigation, the digital medium through which more information could have been transmitted was via messaging to parents’ mobile phones, since the weekly messages were received by the parents of 99% of the IG participants. This is consistent with other studies which have reported greater communication effects when using text messages versus web portals [54]. Accordingly, the use of mobile phones to send short health promotion messages could be an appropriate means of transmitting information in educational interventions. However, additional research is required to clearly determine which is the most effective medium for the transmission of educational information to parents in order to actively involve them in changing their children’s habits. In this study, the parents of schoolchildren showed low attendance at face-to-face meetings, therefore, educational strategies should include non-face-to-face communication that allows parents to learn about and reflect on issues without having to attend meetings, it is necessary to evaluate the effect on behavioral changes in health of these types of strategies and investigate how to adapt them to the conditions of the target population.

In this study, the effect on screen time was observed until the 12 months of follow-up, unlike other community interventions, where the effects on the IG are usually observed earlier and then diminish as follow-up progresses. However, the investigation by Martin et al. found that the effects on the reduction in time in sedentary behaviors were usually still observed at 3 months and could be continued up to 12 months in interventions aimed at adults [36]. Barnett et al. mentioned that the effects on sedentary behaviors of children are usually observable and measurable only in the long term in interventional studies, highlighting that interventions designed specifically to change these behaviors usually have little effect but are significant when they last longer than 6 months [55]. The accumulation of educational messages throughout the follow-up could have had an influence on the actions taken to reduce the time spent in front of screens. However, the effect during follow-up or at the end of the intervention was not seen in the MVPA variable. Therefore, communication strategies should be reinforced to ensure that the PA information provided is reflected in the adjustments toward healthy behaviors of physical activation. As Baranowski and Lytle mentioned, research is required on the ideal exposure time for interventions, the follow-up duration in order to assess the effect’s continuation, and on mediating variables that might influence the effectiveness of interventions over time [56].

Having observed different behaviors in the variables of interest, MVPA and time in front of screens, the importance of addressing physical inactivity and sedentary lifestyle independently is confirmed [57], without expecting that an increase in one necessarily leads to a reduction in the other or vice versa. Emphasis is increasingly being placed on the need to increase the population’s knowledge of the difference between physical inactivity and a sedentary lifestyle, as well as the negative effects that both can have on health, both in the short and long term [58].

Among the strengths of this study is the proposal of a practical strategy to implement an educational intervention conducted in primary schools which involves both children and parents. It is feasible to implement the different means of communication used in the strategy on a larger scale. The results presented reflect the 12-month follow-up, while most studies report shorter follow-up times [50,59,60,61,62]. Finally, as previously published, favorable changes can be obtained in sedentary behaviors, as well as in nutritional status and metabolic parameters, through this strategy [27,28].

Despite the afore mentioned, this study has certain limitations. An individual randomization process was not performed, as randomization was performed by school. Contacting participants within the schools did not make randomization feasible without contamination of the study groups. Additionally, measuring the variables of interest through a questionnaire may not correctly reflect the sedentary activities and PA of participants and resulting data are susceptible to information and memory bias. Social stigmatization of physical inactivity may also have caused an underestimation of screen time or an overestimation of PA because of what is considered as more appropriate behavior. Other weaknesses of the study include the high percentage of losses to follow-up (50%), which is one of the disadvantages of follow-up studies; however, the main losses were due to the fact that the participants did not return the questionnaire that the children took to their parents to answer, and they had to return it to the schools and give it to the researchers. In addition, other sedentary behaviors apart from screen time were not considered. Furthermore, the outcome measurements and exposure to the intervention were reported by the parents and not through reports by the children themselves, which is made difficult by age. Young children usually have trouble quantifying the timeframe of their activities and recalling them objectively [63,64]. Children may be more receptive to intervention and education than parents, and even more so when part of the intervention is conducted within schools. On the other hand, the study sent information to parents through various channels, in person, through the children, over the Internet, and by mobile phones; despite the fact that approximately 43% of the parent participants did not attend any sessions or did not visit the website, it is important to emphasize that the parents who attended the sessions and those who consulted the web the most are presumably the most informed and implement the most changes, in this case, the children decreased the time in front of screens.

Due to the afore mentioned, continued research efforts are required to improve the educational strategies that impact risk behaviors which lead to childhood excess weight and obesity. These investigations should consider more objective parameters for measuring PA and sedentary lifestyle (e.g., accelerometers, inclinometers, pedometers, etc.). Studies that assess the impact of these educational strategies on a larger scale and include the measurement of long-term effects are also needed. Cost-effectiveness studies need to be conducted and evaluated in other cultural, socioeconomic, and geographic contexts. 

Finally, it is important to mention that, although this study took place several years ago, this type of intervention is especially relevant in the current times, when the recent pandemic caused important modifications in the lifestyles of both young and old people around the world, shifting many work and recreational activities into more sedentary alternatives and significantly increasing time spent in front of screens. Due to the significant increase in sedentary behaviors and the increase in the prevalence of childhood obesity, it is necessary to carry out research on interventions aimed at promoting more active and healthy lifestyles. Further research is needed to constantly update data concerning the physical activity of school-age children, adolescents, and adults and the sedentary behavior of these groups, as well as the use of digital media to perform physical activity, such as on-line recorded or live classes, the use of applications with plans structured for physical activity, applications to measure and intervene in times of sedentary lifestyle, to have active pauses in activities in school or at work, etc., in order to update the information to generate or adapt different interventions for specific populations.

## 5. Conclusions

The intervention of multiple face-to-face components (e.g., workshops, museum visits, informative posters, and educational materials) and remote components (websites and text messages to mobile phones) directed toward both parents and schoolchildren reduced the reported daily time that schoolchildren spent in front of screens at a 12-month follow-up. This strategy may be feasible and accessible for promoting changes in sedentary behaviors and to disseminate relevant information on healthy habits among school populations at the community level.

## Figures and Tables

**Table 1:** General characteristics of the participants at baseline.

Characteristics	All (n = 368) Median (IQR)	Intervention (n = 201) Median (IQR)	Control (n = 167) Median (IQR)	*p* *
Sex, (males), n (%)	196 (53.3)	111 (55.2)	85 (50.9)	0.408
Age, (years)	8.0 (7.0, 9.0)	8.0 (7.0, 8.9)	8.1 (7.0, 9.2)	0.347
Weight ^1^, (kg)	29.8 (25.2, 33.9)	29.8 (25.2, 33.5)	29.9 (25.1, 34.6)	0.351
Height ^1^, (cm)	127.2 (120.6, 133.0)	127.4 (120.5, 132.5)	127.1 (120.7, 134.2)	0.381
IMC classification, n (%)				
Normal weight	198 (53.8)	115 (57.2)	83 (49.7)	
Overweight	87 (23.6)	47 (23.4)	40 (23.9)	
Obesity	83 (22.6)	39 (19.4)	44 (26.4)	0.232
Maternal schooling, n (%) n = 364				
Primary/Secondary	65 (17.9)	42 (20.9)	23 (14.1)	
High school/Technical school	148 (40.7)	75 (37.3)	73 (44.8)	
Undergraduate/Postgraduate	151 (41.5)	84 (41.8)	67 (41.1)	0.168
Type of school				
Private	148 (40.2)	73 (36.3)	75 (44.9)	0.094
Public	220 (59.8)	128 (63.7)	92 (55.1)	
Socioeconomic level, n (%) n = 350				
Tertile 1 (Low)	103 (29.4)	63 (32.3)	40 (25.8)	
Tertile 2 (Middle)	126 (36.0)	75 (38.5)	51 (32.9)	
Tertile 3 (High)	121 (34.6)	57 (29.2)	64 (41.3)	0.060
Family integration, n (%) n = 357				
Only father or mother	111 (31.1)	46 (23.7)	65 (39.9)	
Both parents	246 (68.9)	148 (76.3)	98 (60.1)	0.001
Mother’s internet access, n (%) n = 315				
At home	267 (84.8)	140 (82.8)	127 (87.0)	0.307
On cell phone	184 (58.4)	92 (54.4)	92 (63.0)	0.124
At work	169 (53.7)	99 (58.6)	70 (48.0)	0.059
Father’s internet access, n (%) n = 315				
At home	206 (82.1)	116 (80.0)	90 (84.9)	0.317
On cell phone	154 (61.4)	87 (60.0)	67 (63.2)	0.606
At work	179 (71.3)	104 (71.7)	75 (70.8)	0.867
Cell phone possession, n (%)				
Father, n = 261	248 (95.0)	141 (94.0)	107 (96.4)	0.379
Mother, n = 363	323 (89.0)	184 (92.0)	139 (85.3)	0.042
AFMV time (min/week), n = 341	150 (0, 360)	120 (0, 300)	180 (0, 360)	0.331
Did not perform AFMV, n (%)	89 (26.1)	49 (26.1)	40 (26.1)	0.991
Screen time (min/d), n = 367	180 (120, 240)	180 (120, 240)	180 (120, 240)	0.778

^1^ Weight and height adjusted for age and gender by multiple linear regression. * Mann–Whitney *U* test or Pearson χ^2^ test; BMI: body mass index; MVPA: moderate to vigorous physical activity; IQR: interquartile range.

**Table 2:** Change in MVPA and total PA (min/week) at 6- and 12-month follow-ups, according to the study groups.

Group	Baseline Median (IQR)	6 Months Median (IQR)	∆ 0–6 Months Median (IQR)	*p* *
MVPA (min/week)				
Intervention (n = 114)	120 (0, 320)	165 (60, 300)	0 (−90, 120)	0.308
Control (n = 101)	180 (0, 360)	160 (0, 330)	0 (−120, 90)	0.787
*p* **	0.462	0.556	0.541	
				
	Baseline Median (IQR)	12 months Median (IQR)	∆ 0–12 months Median (IQR)	*p* *
Intervention (n = 90)	120 (60, 360)	180 (60, 300)	0 (−120, 135)	0.587
Control (n = 72)	165 (0, 360)	120 (0, 285)	0 (−135, 60)	0.062
*p* **	0.706	0.097	0.085	
TPA (min/week)				
	Baseline Median (IQR)	6 months Median (IQR)	∆ 0–6 months Median (IQR)	*p* *
Intervention (n = 114)	150 (60, 345)	180 (60, 330)	0 (−90, 140)	0.199
Control (n = 101)	180 (0, 360)	180 (60, 360)	0 (−180, 120)	0.886
*p* **	0.679	0.509	0.472	
				
	Baseline Median (IQR)	12 months Median (IQR)	∆ 0–12 months Median (IQR)	*p* *
Intervention (n = 90)	150 (0, 360)	180 (60, 360)	0 (−120, 120)	0.548
Control (n = 72)	180 (0, 360)	120 (30, 300)	0 (−180, 60)	0.110
*p* **	0.855	0.130	0.107	

∆ = change in MVPA or TPA; MVPA: moderate to vigorous physical activity; TPA: total physical activity; IQR: interquartile range; * Wilcoxon test for paired data; ** Mann–Whitney *U* test.

**Table 3:** Change in screen time (min/d) at 6- and 12-month follow-ups, according to the study groups.

Group	Baseline Median (IQR)	6 Months Median (IQR)	∆ 0–6 Months Median (IQR)	p *
Intervention (n = 134)	180 (120, 240)	180 (120, 240)	0 (−60, 60)	0.529
Control (n = 117)	180 (120, 240)	180 (120, 240)	0 (−60, 60)	0.853
*p* **	0.486	0.577	0.766	
				
	Baseline Median (IQR)	12 months Median (IQR)	∆ 0–12 months Mean (CI 95%)	*p* *
Intervention (n = 103)	210 (120, 270)	165 (120, 210)	−33.4 (−53.5, −13.3)	0.001
Control (n = 81)	180 (120, 240)	190 (120, 300)	12.5 (−10.5, 35.6)	0.282
*p* **	0.639	0.016	0.003	

∆ = change in screen time; IQR: interquartile range; CI: confidence interval; * Wilcoxon test for paired data or Student’s *t*-test for dependents. ** Mann–Whitney U test or Student’s *t*-test for independent data.

## Data Availability

The data presented in this study are available on request from the corresponding author. The data are not available publicly.

## Supplementary Materials

The following supporting information can be downloaded at: https://www.mdpi.com/article/10.3390/ijerph20043003/s1, Figure S1: Example of one of the posters that were placed in the participating schools to promote physical activity. Table S1: Change in MVPA (min/week) at 6- and 12-month follow-ups, according to parental exposure to educational components; Table S2: Change in screen time (min/d) at 6- and 12-month follow-ups, according to parental exposure to educational components.
