# Supplementary material for: A Multi-Component Educational Intervention for Addressing Levels of Physical Activity and Sedentary Behaviors of Schoolchildren
Source: Int J Environ Res Public Health. 2023 Feb 9;20(4):3003. doi: 10.3390/ijerph20043003 (PMC9966386; doi:10.3390/ijerph20043003)
Supplement: Supplementary file 1 [file ijerph-20-03003-s001.zip › ijerph-2116084-supplementary.pdf]

Supplementary Figure 1. Example of one of the posters that were placed in the participating schools to promote physical activity

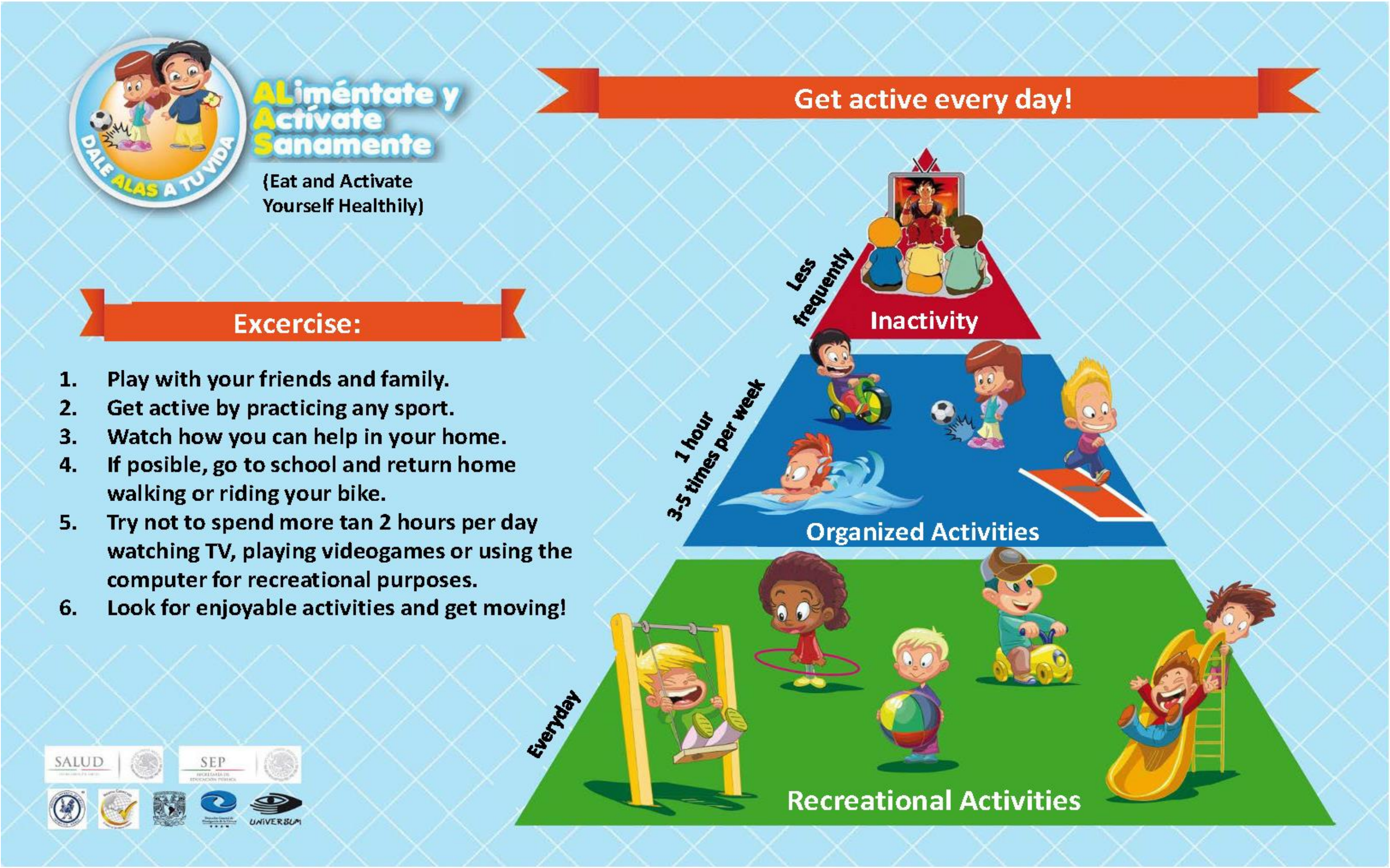

Supplementary Table 1. Change in MVPA (min/wk) at 6 and 12-month follow-ups, according to parental exposure to educational components

Group		Baseline Median (IQR)	6 m Median (IQR)	Δ 0–6 m Median (IQR)	*p**
**Attendance to sessions**					
**Did not attend** (*n* = 38)		110 (0, 240)	170 (60, 300)	60 (-80, 210)	0.259
**Attended at least one session** (*n* = 76)		120 (20, 360)	160 (60, 360)	0 (-90, 120)	0.732
	p*	0.175	0.703	0.418	
		**Baseline Median (IQR)**	**12 m Median (IQR)**	**Δ 0–12 m Median (IQR)**	***p****
**Did not attend** (*n* = 26)		110 (0, 360)	120 (0, 240)	0 (-120, 90)	0.524
**Attended at least one session** (*n* = 64)		120 (0, 360)	180 (120, 360)	0 (-105, 165)	0.324
	p*	0.612	0.078	0.418	
**Consulted the webpage**					
		**Baseline Median (IQR)**	**6 m Median (IQR)**	**Δ 0–6 m Median (IQR)**	***p****
**Did not consult** (*n* = 51)		120 (60, 360)	200 (120, 300)	60 (-90, 150)	0.242
**Consulted the webpage** (*n* = 63)		120 (0, 300)	120 (0, 315)	0 (-90, 120)	0.844
	p*	0.640	0.151	0.427	
		**Baseline Median (IQR)**	**12 m Median (IQR)**	**Δ 0–12 m Median (IQR)**	***p****
**Did not consult** (*n* = 38)		120 (0, 360)	180 (0, 360)	0 (-120, 180)	0.319
**Consulted the webpage** (*n* =52)		135 (42.5, 345)	180 (120, 300)	0 (-120, 120)	0.843
	p*	0.600	0.975	0.428	

Δ = change in AFMV time

MVPA: moderate to vigorous physical activity; IQR: interquartile range

* Wilcoxon test for paired data

** Mann–Whitney *U* test

Supplementary Table 2. Change in screen time (min/d) at 6 and 12-month follow-ups, according to parental exposure to educational components

Group		Baseline<br>Median (IQR)	6 m<br>Median (IQR)	Δ 0–6 m<br>Median (IQR)	*p**
**Attendance to sessions**					
**Did not attend** (*n* = 47)		150 (90, 240)	180 (120, 270)	0 (-60, 120)	0.414
**Attended at least one session** (*n* = 87)		188 (120, 240)	180 (120, 210)	0 (-60, 30)	0.176
	*p***	0.287	0.395	0.195	
		**Basal**<br>**Median (IQR)**	**12 m**<br>**Median (IQR)**	**Δ 0–12 m**<br>**Mean ± SD**	***p****
**Did not attend** (*n* = 31)		180 (120, 270)	180 (120, 240)	-7.7 ± 78.1	0.420
**Attended at least one session** (*n* = 72)		210 (120, 240)	150 (115, 180)	-44.4 ± 110.5	0.001
	*p***	0.891	0.115	0.097***	
**Consulted the webpage**					
		**Basal**<br>**Median (IQR)**	**6 m**<br>**Median (IQR)**	**Δ 0–6 m**<br>**Median (IQR)**	***p****
**Did not consult** (*n* = 66)		155 (90, 240)	170 (120, 210)	-22.5 (-60, 60)	0.657
**Consulted the webpage** (*n* = 68)		180 (130, 231)	180 (120, 240)	0 (-60, 60)	0.666
	*p***	0.141	0.159	0.815	
		**Basal**<br>**Median (IQR)**	**12 m**<br>**Median (IQR)**	**Δ 0–12 m**<br>**Mean ± SD**	***p****
**Did not consult** (*n* = 44)		210 (105, 270)	168 (115, 240)	-17.3 ± 103.5	0.539
**Consulted the webpage** (*n* =59)		200 (140, 240)	165 (120, 210)	-45.4 ± 101.6	<0.001
	*p***	0.735	0.552	0.171***	

Δ = change in screen time

IQR: interquartile range; *SD*: Standard deviation

* Wilcoxon test for paired data

** Mann–Whitney *U* test

*** Student's *t*-test for independent data.
